# Selection of vaccine strains for serotype O foot-and-mouth disease viruses (2007–2012) circulating in Southeast Asia, East Asia and Far East

**DOI:** 10.1016/j.vaccine.2017.10.099

**Published:** 2017-12-18

**Authors:** Mana Mahapatra, Sasmita Upadhyaya, Sharie Aviso, Aravindh Babu, Geoff Hutchings, Satya Parida

**Affiliations:** a The Pirbright Institute, Ash Road, Pirbright, Surrey GU24 ONF, UK; b School of Veterinary and Biomedical Sciences, Murdoch University, Murdoch 6150, Australia

**Keywords:** Southeast Asia, East Asia, Far East, FMD, Serotype O, Vaccine strain selection

## Abstract

Foot-and-mouth disease (FMD) is endemic in Southeast Asia (SEA) and East Asia with circulation of multiple serotypes and multiple genotypes within each serotype of the virus. Although countries like Japan and South Korea in the Far East were free of FMD, in 2010 FMD serotype O (O/Mya-98) outbreaks were recorded and since then South Korea has experienced several FMD outbreaks despite regular vaccination. In this study a total of 85 serotype O FMD viruses (FMDV) isolated from 2007 to 2012 from SEA, East Asia and Far East were characterized by virus neutralisation tests using antisera to four existing (O/HKN/6/83, O/IND/R2/75, O/SKR/2010 and O/PanAsia-2) and one putative (O/MYA/2009) vaccine strains, and by full capsid sequencing. Serological studies revealed broad cross-reactivity with the vaccine strains; O/PanAsia-2 exhibited a good match with 95.3%, O/HKN/6/83 with 91.8%, O/IND/R2/75 with 80%, and the putative strain O/MYA/2009 with 89.4% isolates employed in this study. Similarly O/PanAsia-2 and O/IND/R2/75 vaccines showed a good match with all eight viruses belonging to O-Ind-2001d sublineage whereas the vaccines of O/Mya-98 lineage, O/MYA/2009 and O/SKR/2010 exhibited the lowest match indicating their unsuitability to protect infections from O-Ind-2001d viruses. A Bayesian analysis of the capsid sequence data indicated these circulating viruses (n = 85) to be of either SEA or Middle East-South Asian (ME-SA) topotype. The ME-SA topotype viruses were mainly detected in Lao PDR, Vietnam, Myanmar and Thailand reflecting the trade links with the Indian subcontinent, and also within the SEA countries. Implications of these results in the context of FMD control in SEA and East Asian countries are discussed.

## 1 Introduction

Foot-and-mouth disease (FMD) is a highly contagious disease of domestic and wild cloven hooved animals across the world. It is endemic in Africa, Middle-East and Asia. The causative agent, FMD virus (FMDV) is a single stranded positive sense RNA virus (genus *Aphthovirus*, family *Picornaviridae)* and exists as seven immunologically distinct serotypes, O, A, C, Asia 1, SAT (Southern African Territory) 1, 2 and 3, each with a wide spectrum of antigenically distinct subtypes [1], [2]. Serotype C was last reported in Brazil and Kenya in 2004 [1], [3] and is probably now extinct. The RNA genome is enclosed in a capsid and encodes for four structural proteins (VP1-4) and 8 non-structural proteins. Sixty copies of the four structural proteins (VP1-4) form the capsid; VP1-3 are exposed on the surface that contain neutralising epitopes whilst the VP4 is internal.

Southeast Asia (SEA), East Asia and Far East have a large population of FMDV susceptible livestock, mainly cattle, pigs and water buffaloes. Regular outbreaks have been reported in countries in SEA: Cambodia, Lao PDR, Malaysia, Myanmar, Thailand, Vietnam, and in East Asia: Mongolia, Hong Kong and China [4]. Indonesia, Singapore and Brunei have remained FMD-free without vaccination. Similarly the Philippines has not reported an outbreak since 2005, and was declared FMD-free without vaccination by OIE in 2011. FMD free countries like Japan and South Korea in the Far East experienced FMD outbreaks in 2010 and since then South Korea has experienced several outbreaks, almost every year. More than 60% of FMD outbreaks in SEA and East Asia are caused by serotype O [1]. Historically the main serotype O topotypes found in SEA and East Asia are Cathay and SEA. In addition another topotype from the Middle East, ME-SA (PanAsia strain) caused extensive outbreaks in Japan, South Korea, China, Taiwan and Russia during 1999–2002 [5], [6], [7], [8] and continues to circulate in the SEA countries.

The predominant SEA topotype strain, O/SEA/Mya-98 was mainly restricted to SEA countries until 2010 (Table 2). In 2010–2011 this strain caused devastating outbreaks in FMD free countries, Japan and South Korea [3], [9], [10], [11], [12], [13] resulting in high economic losses. O Manisa vaccine was used to control the disease, however full clinical protection was not observed in South Korea. In spite of a compulsory vaccination campaign, clinical disease has been reported in South Korea every year since 2014 [15, WRLFMD reports; http://www.wrlfmd.org/] which raises questions about the effectiveness of vaccination in pigs. Amongst many possible reasons, a non-matching vaccine could be important [15]. Indeed vaccine matching work carried out at World Reference Laboratory (WRL), Pirbright using South Korean viruses from the years 2010 and 2011 indicated that only 60% (three out of five) of the isolates matched with O Manisa vaccine in-vitro [WRLFMD report; http://www.wrlfmd.org/]. Recently another strain of ME-SA topotype, O-Ind-2001d originating from the Indian subcontinent has spread to new areas and caused outbreaks thereby complicating the epidemiological situation. Initially these O-Ind-2001d viruses were restricted to the Indian subcontinent [16], however they have been detected in the Middle East and North Africa since 2013 [17], [18]. These O-Ind-2001d viruses has also been detected in Lao PDR since 2015 and have now spread to other neighbouring countries like Vietnam, Myanmar, Thailand and South Korea (Table 1), possibly by trade links with Indian subcontinent, and animal movement between SEA countries [19], [20].They have also been detected in Russia in 2016 and China in 2017 emphasizing the importance of a matching vaccine for use in FMD control programmes. To our knowledge there is no published report on the FMD vaccine strain selection for SEA, East Asia and Far East. Therefore this study was designed to carry out a systematic study to select an appropriate serotype O vaccine strain for use in the SEA and East Asian countries for FMD control.

**Table 1:** Topotypes/lineages circulating in SEA countries from 2005 to 2017. Additionally, SEA (Mya-98) viruses have been detected in Russia and China in 2010; ME-SA (PanAsia) in Russia (2010), China (2005, 2011) and Kazakstan (2007, 2010–12); ME-SA (Ind-2001d) in Russia (2016) and China (2017).

Country/Topotype	Cathay	SEA (Mya-98)	ME-SA (PanAsia)	ME-SA (Ind-2001d)
Cambodia	–		2006, 2008, 2010, 2012–13, 2015	–
Demo. R. Korea	–	2011	–	–
Hong Kong	2005–11, 2013–16	2011–12	–	–
Japan	–	2010	–	–
Lao PDR	–	2007–2010, 2013, 2016	2006, 2010–12	2015
Malaysia	–	2005, 2007, 2009–12, 2016	2005–6, 2009	–
Mongolia	–	2010, 2015	2014	–
Myanmar	–	2006, 2008–10, 2015	–	2016
Phillipines	2005	–	–	–
South Korea	–	2010–11, 2014–16	–	2017
Taiwan	2009, 2013	2012	–	–
Thailand	2005, 2012	2005, 2007, 2011, 2016	2011, 2015	2016
Vietnam	2005, 2008, 2016	2005–06, 2010, 2014–16	2005, 2008, 2010–14	2015–16

## 2 Materials and methods

### 2.1 Cells, viruses and bovine post-vaccinal sera (BVS)

Eighty-five serotype O viruses from SEA, East Asia and Far East submitted to the WRL FMD at Pirbright and two vaccine strains from Middle East and India were used in this study (Supplementary Table 1). Three are the vaccine strains O/HKN/6/83, O/PanAsia-2 (O/TUR/5/2009) and O/IND/R2/75 that were originally isolated from Hong Kong, Turkey and India in 1983, 2009 and 1975, respectively. The other 84 viruses were isolated over a six-year period between 2007 and 2012. These originated from eleven countries; Cambodia (n = 7), Democratic Republic of Korea (n = 1), Hong Kong (n = 4), Japan (n = 1), Lao PDR (n = 9), Malaysia (n = 8), Mongolia (n = 1), Myanmar (n = 7), South Korea (n = 6), Thailand (n = 16) and Vietnam (n = 25). In addition, eight serotype O viruses (isolated over a four year period between 2009 and 2012 from the Indian subcontinent) belonging to O-Ind-2001d lineage were also included in this study making a total of 95 viruses (Supplementary Table 1). The samples from SEA countries were derived from either cattle (n = 47), buffalo (n = 9) or pig (n = 27) epithelial tissues except two viruses from Cambodia whose host species is not known (Supplementary Table 1). All eight isolates from the Indian subcontinent were of cattle origin. All the samples were initially grown in primary bovine thyroid cells (BTY) with subsequent two to three passages in IB-RS2 (pig kidney) cells. Stocks of virus were prepared by infecting IB-RS2 cell monolayers and were stored as clarified tissue culture harvest material at −70 °C until required.

Five bovine anti-FMDV post-vaccination sera (BVS) were used in the study. Two of these, namely O/IND/R2/75 and O/PanAsia-2 have been described in detail previously [21], [22]; one antisera, O/SKR/2010 (existing vaccine of O/SEA/Mya-98 lineage) was procured from MSD Animal Health, Germany whereas the other two were generated in this study. The antisera were raised against the Cathay topotype vaccine strain, O/HKN/6/83 and the putative strain, O/MYA/2009 of O/SEA/Mya-98 lineage, in cattle at Pirbright as described previously [23] by administering inactivated, purified 146S FMDV particles in ISA-206 adjuvant. The animals were boosted on 21-day post-vaccination and bled one week later. For each antigen, a pool of sera from five animals was used in the serological tests. The homologous neutralising antibody titres of each pooled serum were in the range of log_10_ 2.58–3.27 (data not shown).

### 2.2 Antigenic characterization by two-dimensional virus-neutralisation test (2D-VNT)

The 2D-VNT test was carried out using the pooled BVS according to Rweyemamu et al. [24]. Antibody titres were calculated from regression data as the log_10_ reciprocal antibody dilution required for 50% neutralisation of 100 tissue culture infective units of virus (log_10_SN_50_/100 TCID_50_). The neutralising antigenic relationship of viruses is given by the ratio: ‘r_1_’ = neutralising antibody titre against the heterologous virus/neutralising antibody titre against the homologous virus. The serological relationship between two viruses in the range ‘r_1_’ = 0.3–1.0 are indicative of a reasonable level of cross protection whereas values less than 0.3 indicate dissimilar strains and the need to acquire, or develop, a new vaccine strain [25]. All the tests were carried out in duplicates, and repeated at least twice, and average values from at least two tests were used for subsequent analysis.

### 2.3 RNA extraction, RT-PCR, nucleotide (nt) sequencing and analysis of the sequence data

For generating the nt sequences of the capsid coding region (P1) of the viruses, RNA extraction, reverse transcription (RT), polymerase chain reaction (PCR), sequencing, sequence analysis and assembling, and alignment were performed as described previously [26]. Nt sequences of the viruses were aligned using the CLUSTAL X multiple sequence alignment program [27] and the predicted amino acid (aa) sequences were translated using BioEdit 7.0.1 [28]. The alignments were used to construct distance matrices using the Kimura 2-parameter nucleotide substitution model [29] as implemented in the program MEGA 6.0 [30].

Using jModelTest [31] and MEGA [30], General time reversal (GTR) model with combination of gamma distribution and proportion of invariant sites (GTR+G+I) was determined to be the most suitable nucleotide substitution model for the complete P1 nucleotide sequences of the SEA type O viruses. Bayesian analysis was performed using the BEAST software package v1.8.4 [32]. In BEAUti v1.8.4, the ages of the viruses were defined by the date of sample collection and the analysis used GTR+G+I model to describe rate heterogeneity among sites. Variations in substitution rate among branches were evaluated by comparing four different clocks in BEAST. The maximum clade credibility (MCC) phylogenetic tree was inferred using the Bayesian Markov Chain Monte Carlo (MCMC) method followed by a Bayes factor analysis in TRACER version 1.6 [33] to determine the best-fit model resulting in the selection of an uncorrelated exponential relaxed molecular clock. Tree Annotator program in BEAST was used to obtain the evolutionary tree and FigTree program 1.4.2 was used to view the trees.

### 2.4 Statistical analysis

The statistical analysis was carried out using Minitab 17 software.

## 3 Results and discussion

### 3.1 Antigenic characterization of the serotype O viruses circulating in SEA, East Asia and Far East

In SEA and East Asia there are a few established serotype O vaccine strains (e.g. O/HKN/6/83, O 4174, O 4625, O 3039, O TAW), mainly developed from previous viruses available for use in the region. In addition serotype O Cathay-like virus vaccines (e.g. O Taiwan 97, O Philippine 97, O 1685 or Russia 95) are also available for vaccinating pigs (http://www.wrlfmd.org/ref_labs/ref_lab_reports/OIE-FAO%20FMD%20Ref%20Lab%20Network%20Report%202011.pdf). There is a locally produced serotype O vaccine of PanAsia origin in China (China 1999) and a Cathay-like virus vaccine (Os99) for use in pigs, however use of these vaccines are mainly restricted to China. Though O/Mya-98 has been circulating in the SEA region for nearly two decades, in 2010 a Mya-98 vaccine strain was selected and developed as a vaccine (O/MYA98/BY/2010) by Lanzhou Veterinary Research Institute (LVRI), China. China uses about 1.5 billion doses of vaccine per year in a six-monthly vaccination strategy, however no reports detailing the efficacy of these vaccines are currently available (http://www.wrlfmd.org/ref_labs/ref_lab_reports/OIE-FAO%20FMD%20Ref%20Lab%20Network%20Report%202012.pdf). South Korea also adapted a Mya-98 virus isolated from South Korea to develop as a vaccine and tested in pigs [34]. Similarly, Thailand uses a locally produced serotype O vaccine, O/TAI/189/87 and recently developed a vaccine of Mya-98 lineage. Though a large number of vaccines have been made over the years no published information is available about the efficacy of these locally produced vaccines. In addition the suitability of these vaccines to be used in FMD control programmes of other SEA/East Asia/Far East countries is yet to be established. Therefore four internationally established vaccines and one putative strain originating from the region covering all the three circulating topotypes were evaluated in this study.

The cross-reactivity of 85 serotype O viruses from SEA, East Asia and Far East employed in this study was measured by 2D-VNT using BVS against five vaccines. Out of these two vaccines, O/HKN/6/83 and O/SKR/2010 are vaccines originating from the region. The vaccine strains, O/PanAsia-2, originating from the Middle East, and O/IND/R2/75, originating from the Indian subcontinent were also included to test their suitability to be used as a vaccine in the SEA region. In addition, antisera against the putative strain, O/MYA/2009 belonging to the predominant topotype, O/SEA/Mya-98 was also included in this study (Supplementary Table 1). O1/Manisa vaccine was not included as it has not provided sufficient protection for South Korean serotype O outbreaks, and has also been shown to produce relatively lower level of neutralising antibodies in pigs against viruses of Mya-98 and PanAsia lineage isolated from SEA [34].

**Fig. 1:**
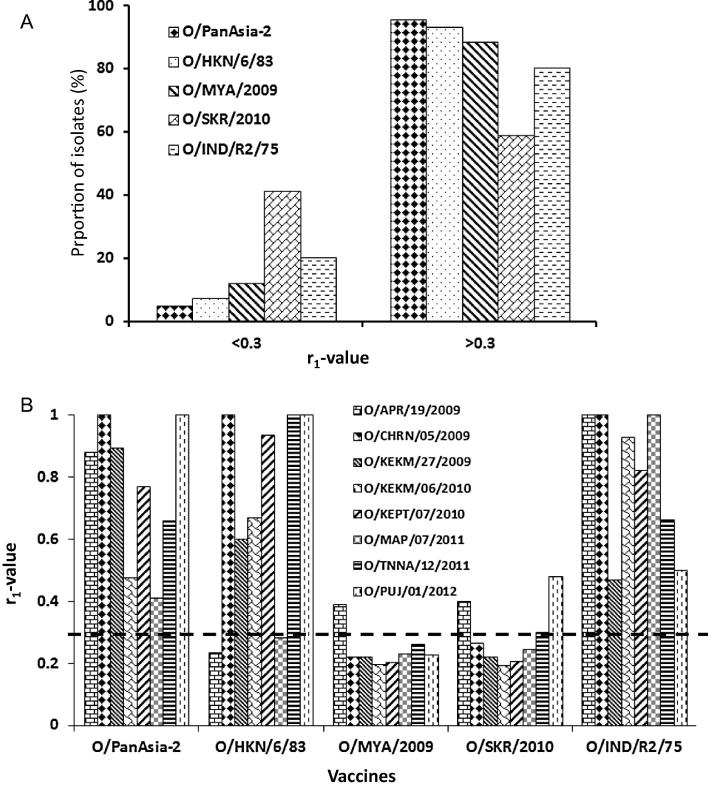
(A) Proportion of serotype O isolates (%) exhibiting antigenic relationship (r_1_) values <0.3 and >0.3 against five post-vaccinal bovine antisera. (B) Antigenic relationship (r_1_) values of serotype O-Ind-2001d viruses against five post-vaccinal bovine antisera. The horizontal dotted line indicates the cut-off value of 0.3, above which the vaccine is considered to be a good match.

The vaccine matching results generated in this study indicates the serotype O vaccines to be broadly cross-reactive (Fig. 1A). All the vaccines except O/SKR/2010 exhibited in-vitro match with more than 80% viruses tested in this study. According to the in-vitro antigenic matching results based on the viruses from the three different topotypes, O/PanAsia-2 vaccine reacted with more of the viruses of O/ME-SA/PanAsia origin, followed by O/IND/R2/75 (Table 2). Similarly O/MYA/2009 vaccine reacted with more viruses from the O/SEA/Mya-98 lineage followed by O/PanAsia-2 and O/HKN//6/83 vaccine (Table 2). Interestingly three vaccines, O/HKN/6/83, O/PanAsia-2 and O/MYA/2009 were a good match with all the Cathay topotype viruses (five out of five) employed in this study. As a whole, O/PanAsia-2 vaccine strain appears to be the best vaccine exhibiting a good match with over 95% viruses employed in this study (Fig. 1A and Table 2) followed by three vaccines in the order O/HKN/6/83, O/MYA/2009 and O/IND/R2/75 (Fig. 1A and Table 2). The least cross-reactive vaccine was O/SKR/2010 matching with only 58% viruses employed in this study (Fig. 1A and Table 2) indicating it is less suitable to be used as a vaccine for FMD control in the region. In a vaccine challenge experiment involving pigs vaccinated with O/SKR/2010 vaccine no detectable amount of neutralising antibody was observed on 21 days post-vaccination (dpv) against O/ME-SA/PanAsia strain while only 60% animals exhibited neutralising antibody above 1:16 on 49 dpv and none of the animals was protected upon challenge with an O/ME-SA/PanAsia virus [34] indicating this vaccine not to be broadly cross-protective. However, high antigen payload vaccines have been shown to compensate poor vaccine match in serotype O [35] and A viruses [36]. The capsid sequences of this vaccine strain (O/SKR/2010) and the non-matching viruses were analysed further to understand the molecular basis of the antigenic mis-match, however no obvious antigenically critical amino acid substitutions were observed (data not shown).

**Table 2:** Proportion (%) of viruses of different topotypes exhibiting antigenic relationship (r_1_) values above 0.3 (good match) against five post-vaccinal bovine antisera used in this study. The lineages are shown in parenthesis wherever applicable. ME-SA: Middle East-South Asia; SEA: Southeast Asia.

Vaccine/topotype	O/PanAsia-2	O/HKN/6/83	O/MYA/2009	O/SKR/2010	O/IND/R2/75
ME-SA (PanAsia)	96.43	85.71	75.00	46.43	89.29
ME-SA (Ind-2001d)	100	75	12.5	25.00	100
SEA (Mya-98)	94.23	94.23	96.15	69.23	78.85
Cathay	100	100	100	20	40
Total	95.29	91.76	89.41	58.82	80.00

**Fig. 2:**
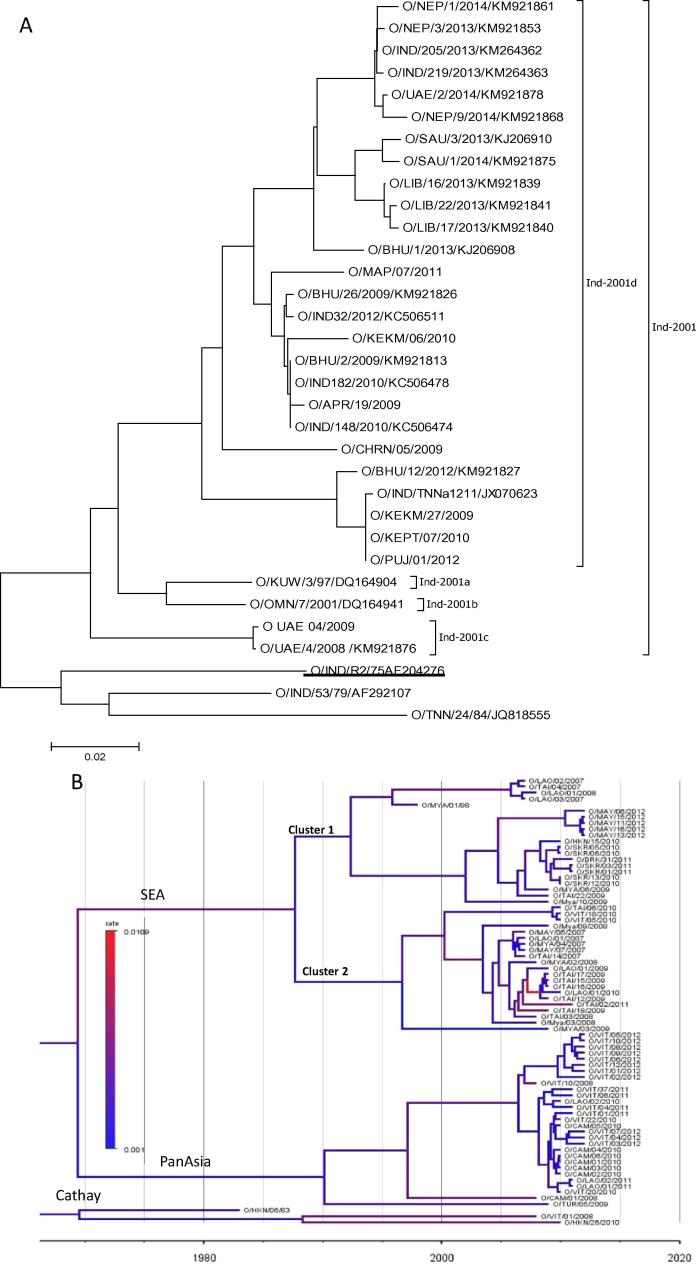
(A) Neighbour joining phylogenetic tree (VP1) showing viruses of O-Ind-2001d sublineage. The Indian vaccine strain used in this study is underlined (B) Bayesian phylogenetic tree of the Southeast Asian serotype O viruses. The sequences retrieved from GenBank contain the accession numbers against each isolate. The topotypes/lineages are shown on the respective branches.

The O-Ind-2001d viruses have been circulating in SEA since 2015 and has spread to the Far East (South Korea) recently (Table 1), and could pose a threat to the livestock industry in future [20]. To the best of our knowledge there is no published report of a matching vaccine that could protect against outbreaks of O-Ind-2001d viruses. Therefore as a first step we have measured the antigenic relationship of the five vaccine strains employed in this study with eight viruses of O-Ind-2001d sub-lineage (Fig. 2A) isolated from India during 2009–2012 [21]. O/PanAsia-2 and O/IND/R2/75 vaccines were a good match with all the O-Ind-2001d viruses whereas O/HKN/6/83 matched with 75% of the O-Ind-2001d viruses (Fig. 1B). The vaccines of O/Mya-98 lineage, O/MYA/2009 and O/SKR/2010 exhibited the lowest in-vitro protection (with only 12.5% and 25% isolates, respectively) indicating they are less suitable to protect infections from O-Ind-2001d viruses. This indicated that the existing serotype O vaccines (O/PanAsia-2 and O/IND/R2/75) should be able to provide protection against O-Ind-2001d viruses.

### 3.2 Genetic characterisation of the serotype O viruses circulating in SEA, East Asia and Far East

The full capsid sequences of 75 SEA, East Asia and Far East viruses were generated in this study. The capsid coding sequences of six more viruses (Supplementary Table 1) were reported in our previous studies [22], [37]. The capsid sequences of four isolates (O/VIT/09/2008, O/MOG/03/2010, O/VIT/11/2012 and O/VIT/13/2012) used for antigenic characterisation in this study could not be generated either because of problems in amplifying the capsid encoding sequences or having ambiguities at more than 10 nt positions that could not be resolved even after repeated attempts. In addition, 24 capsid sequences available in GenBank were also included in the analysis making a total of 105 sequences. All the sequences were 2202 nt long. Compared to the oldest established vaccine strain of the region, O/HKN/6/83, there was 12.3% (O/VIT/01/2008) to 19.5% (O/SKR/01/2011) variation at nt level and 2.86% (O/VIT/01/2008) to 6.54% (O/LAO/01/2008, O/MYA/10/2009, O/JPN/01/2010) variation at aa level. Similarly, compared to the oldest viral sequence of Mya-98 lineage employed in our analysis, O/MYA/1/98, the variation at nt level was 4.88% (O/MYA/03/2009) to 20.2% (O/VIT/01/2008), and 1.77% (O/LAO/02/2007) to 8.17% (O/HKN/26/2010) at aa level. Analysis of the sequence dataset by the Z-test (using MEGA) for evidence of evolutionary selection did not exhibit significant difference (P ≥ 1.0) which probably explains why the serotype O vaccines are broadly cross-reactive. This observation is in line with similar studies involving serotype O viruses from other geographical areas [21].

The 81 full capsid sequences generated in this study/our previous study were used for Bayesian phylogenetic analysis. The results indicated three different topotypes (ME-SA, SEA and Cathay) of FMDV serotype O viruses circulating in the region (Fig. 2B). Within the SEA topotype, viruses of Mya-98 lineage formed two distinct clusters (Fig. 2B). This could indicate change in antigenicity of the viruses in future as has been exemplified by emergence of antigenic variants within A-Iran-05 viruses in the Middle East [38]. Therefore close monitoring of the outbreaks and regular surveillance is necessary for the control of the disease in the region. Interestingly the closest virus to the South Korean virus (collected in November-December 2010) is the O/HKN/15/2010 virus (collected in February 2010) from Hong Kong (Fig. 2B) with 0.82% and 0.68% differences at nt and aa level, respectively. The O/ME-SA/PanAsia viruses are mainly circulating in Cambodia, Japan, Lao PDR, Malaysia, South Korea and Vietnam. The Cathay topotype viruses are mainly restricted to Hong Kong and Vietnam in the recent years although these viruses have been detected in Phillipines in 2005, Thailand in 2012 and Taiwan in 2009 and 2013. However they may still be circulating in the region as all the outbreaks are neither reported nor investigated.

From the Bayesian analysis, using an uncorrelated exponential relaxed molecular clock, the rate of substitution of all the nt changes in the capsid coding region of the serotype O viruses from SEA was estimated to be 3.17 × 10^−3^/site/year (95% HPD 2.13 × 10^−3^–4.33 × 10^−3^). This is similar to our previous report for Indian serotype O FMD viruses (1.78.7x10^3^ substitutions/site/year) [8] and also by others in serotype O FMD viruses [39], [40], [41], [42], [43], [44].

In conclusion, the serotype O vaccine strains used in this study are a good match with the circulating field isolates in SEA, East Asia and Far East; O/PanAsia-2 reacting to the most viruses followed by O/HKN/6/83, O/MYA/2009 and O O/IND/R2/75. This indicates that serotype O vaccine strains are broadly cross-reactive and, therefore could be used as a vaccine to control the disease in the endemic countries in the region. There is always a risk of emergence of antigenic variants or introduction of new lineages/strains of the viruses to the SEA countries as exemplified by the introduction of O-Ind-2001d viruses in Lao PDR in 2015, and its subsequent spread to other SEA countries. Findings of this study reveal that the existing serotype O vaccines (O/PanAsia-2 and O/IND/R2/75) would be able to provide protection against O-Ind-2001d viruses. The Progressive Control Pathway (PCP) for FMD as described by OIE/FAO is mainly to assist endemic countries to reduce the impact of FMD progressively and has six (0–5) different stages. Currently, the SEA countries are in different stages of PCP-FMD and would benefit from a robust matching vaccine to move forward in the program. In addition, epidemiology including close monitoring of the outbreak strains in the region along with regular vaccine matching studies is critical to evaluate the suitability of the vaccine strains for use in FMD control programmes. In order to progress on PCP-FMD for the region an intense co-ordinated regional approach including implementation of regional control measures, stronger diagnostic capacity and field veterinary services, improved bio-security measures and control of cross-border animal movement are essential.
