# Supplementary material for: Selection of vaccine strains for serotype O foot-and-mouth disease viruses (2007–2012) circulating in Southeast Asia, East Asia and Far East
Source: Vaccine. 2017 Dec 18;35(51):7147–53. doi: 10.1016/j.vaccine.2017.10.099 (PMC5720463; doi:10.1016/j.vaccine.2017.10.099)
Supplement: Supplementary data 1 [file mmc1.docx]

Supplementary Table 1: List of serotype O FMD viruses used in this study. ME-SA: Middle East-South Asia; SEA: Southeast Asia; NK: not known; ND: not done. The virus sequences with accession numbers with an * (n=22) has been generated in this study and the virus sequences with accession numbers without an * (n=8) were either generated in our previous study [22] or extracted from GenBank. The remaining 53 capsid sequences have been submitted to GenBank and are awaiting accession number.
